# Glucuronides of phytoestrogen flavonoid enhance macrophage function via conversion to aglycones by β‐glucuronidase in macrophages

**DOI:** 10.1002/iid3.163

**Published:** 2017-05-08

**Authors:** Atsushi Kaneko, Takashi Matsumoto, Yosuke Matsubara, Kyoji Sekiguchi, Junichi Koseki, Ryo Yakabe, Katsuyuki Aoki, Setsuya Aiba, Kenshi Yamasaki

**Affiliations:** ^1^ Tsumura Research Laboratories Tsumura & Co. Ami‐machi, Inashiki‐gun, Ibaraki Japan; ^2^ Analytical and Pharmaceutical Technology Research Center Tsumura & Co. Ami‐machi, Inashiki‐gun, Ibaraki Japan; ^3^ Department of Dermatology Tohoku University Graduate School of Medicine Aoba‐ku, Sendai, Miyagi Japan

**Keywords:** β‐glucuronidase, estrogen receptor, macrophages

## Abstract

**Introduction:**

Flavonoids are converted to inactive metabolites like glucuronides in the gut, and circulate mainly as glucuronides in blood stream, resulting in low concentrations of active aglycones in plasma. It is therefore unclear how oral flavonoids exert their effects in tissues. We recently reported the plasma pharmacokinetics of some flavonoids and suggested the possibility that the absorbed flavonoids modified macrophage functions leading to enhance bacterial clearance. We aimed to confirm their pharmacological profiles focusing on tissue macrophages.

**Methods:**

Pseudoinfection was induced by intradermal injection of FITC‐conjugated and killed *Staphylococcus aureus* into the ears of mice treated with or without genistein 7‐*O*‐glucuronide (GEN7G, 1 mg/kg, i.v.). FACS analysis was performed on single cell suspensions dispersed enzymatically from the skin lesions at 6 h post pseudoinfection to evaluate phagocytic activities of monocytes/macrophages (CD11b^+^Ly6G^−^) and neutrophils (CD11b^+^Ly6G^+^). Phagocytosis of the FITC‐conjugated bacteria by four glucuronides including GEN7G was evaluated in cultures of mouse macrophages.

**Results:**

After GEN7G injection, genistein was identified in the inflamed ears as well as GEN7G, and the phagocytic activity of CD11b^+^Ly6G^−^ cells was increased. GEN7G was converted to genistein by incubation with macrophage‐related β‐glucuronidase. Macrophage culture assays revealed that GEN7G increased phagocytosis, and the action was dampened by a β‐glucuronidase inhibitor. Binding of aglycones to estrogen receptors (ERs), putative receptors of flavonoid aglycones, correlated to biological activities, and glucuronidation reduced the binding to ERs. An ER antagonist suppressed the increase of macrophage function by GEN7G, whereas estradiol enhanced phagocytosis as well.

**Conclusions:**

This study suggests a molecular mechanism by which oral flavonoids are carried as glucuronides and activated to aglycones by β‐glucuronidase in tissue macrophages, and contributes to the pharmacological study of glucuronides.

## Introduction

A growing number of studies have been performed over decades for pharmacological verification of the effects of flavonoids, which are believed to be beneficial for good health 1, 2, 3, 4. Flavonoid aglycones exert multiple effects such as anti‐oxidation, ‐inflammation, and ‐cancer in vivo and in vitro. Flavonoid glucuronides can be potent precursors of the biologically active aglycones but do not always show biological activities similar to aglycones 5, 6, 7. However, due to their metabolism in the body, aglycones comprise a very small concentration in the plasma of human and animals after oral administration, and glucuronides and/or sulfate‐conjugates are the main circulating metabolites of flavonoids 8, 9, 10, 11, 12, 13. Due to chemical and structural differences between aglycones and glucuronides in terms of size, solubility, and polarity, flavonoid glucuronides are generally understood to be unable to pass through the cellular membranes of the lipid bilayer and to cross the vascular lumen to extravascular spaces. The path to an active aglycone, β‐glucuronidase, which exists in macrophages and deconjugates flavonoid glucuronides to active aglycones, has been elucidated. The function of β‐glucuronidase has been actively demonstrated in the field of cardiology using quercetin, which has strong anti‐oxidant and ‐inflammation effects 6, 7. Because various types of flavonoids show biological effects on immune responses, the metabolism of flavonoids is thought to play biological roles in detoxification and excretion of foreign substances.

Jumihaidokuto (JHT) is a pharmaceutical‐grade traditional Japanese medicine prescribed to patients with inflammatory dermatoses. We previously demonstrated the blood pharmacokinetics of the flavonoids identified in JHT, showing that various types of flavonoid glucuronides appeared in the plasma of rats given JHT orally 13. In particular, genistein 7‐*O*‐glucuronide (GEN7G), liquiritigenin 7‐*O*‐glucuronide (LQG7G), liquiritigenin 4′‐*O*‐glucuronide (LQG4′G), and hesperetin 7‐*O*‐glucuronide (HPT7G) were identified in the plasma, although concentrations of their aglycones were extremely low. We moreover found that JHT suppressed *Propionibacterium acne*‐induced dermatitis by modulating macrophage functions 14. These observations suggested a relationship between the effect of JHT on macrophage activation and the metabolism of flavonoids.

In this study, we hypothesized that the flavonoids generated by JHT administration augment the macrophage functions in skin infection. We first examined the pharmacological profiles of GEN7G focusing on interaction with macrophages in vivo and in vitro. The results of the present study showed that flavonoid glucuronides play roles as a carrier and a precursor of bioactive aglycones, and that flavonoids can enhance macrophage functions through agonistic potentials on the nuclear estrogen receptor (ER).

## Materials and Methods

### Test compounds

Genistein, liquiritigenin, and hesperetin were purchased from Wako Pure Chemical Industries (Osaka, Japan). Genistein 7‐*O*‐glucuronide (GEN7G) and hesperetin 7‐*O*‐glucuronide (HPT7G) were purchased from Toronto Research Chemical Industries (Toronto, ON). Liquiritigenin 4′‐*O*‐glucuronide (LQG4′G) and liquiritigenin 7‐*O*‐glucuronide (LQG7G) with purities high enough to be evaluated in biological tests were supplied by Tsumura & Co (Tokyo, Japan). The chemical structures of these flavonoids are shown in Supplemental Figure S1. Estradiol (Sigma–Aldrich, St. Louis, MO) was used as a reference.

### Pseudoinfection model

Male ICR mice were purchased from Charles River Laboratories Japan (Kanagawa, Japan), allowed housed at a temperature of 23 ± 3°C, relative humidity of 50 ± 20%, and a 12 h light:12 h dark cycle with lights on from 07:00 to 19:00 h daily with free access to water and standard laboratory food. All experimental procedures were approved by and conducted according to the guidelines of the experimental animal ethics committees of Tsumura & Co.

Particles of FITC‐conjugated and killed *Staphylococcus aureus* (FITC‐*S. aureus*) (Molecular Probes, Eugene, OR) were suspended in saline, and injected intradermally into the dorsal side of the ear at 67 µg/10 µl/site using a microsyringe (Hamilton Co., Reno, NV) under inhaled isoflurane anesthesia 14.

### Measurement of genistein and GEN7G in pseudoinfection model

GEN7G was injected intravenously into mice at 1 mg/kg in saline immediately and 3 h after pseudoinfection. The mice were exsanguinated 6 h after pseudoinfection, followed by dissection of the ears, and their homogenization in 50 mmol/L Tris–HCl (pH 8.3) containing 20 mmol/L of saccharic acid 1,4‐lactone (Santa Cruz Biotech., Santa Cruz, CA), which is a β‐glucuronidase inhibitor. The samples were stored at −80°C until measurement of genistein and GEN7G by LC‐MS/MS as described below 13. For quantification of both compounds in the homogenized tissues, a portion of the homogenized tissue was diluted with water, mixed with an internal standard (niflumic acid; Sigma–Aldrich), and the solid phase was extracted by using a Sep‐Pak Vac (Waters, Milford, MA). The cartridge was conditioned with 2 mL of methanol and water before use. The diluted solution was loaded into the preconditioned columns, washed with 0.8 mL of water, and eluted with 0.8 mL of methanol. The eluent was collected and dried using a centrifugal evaporator and concentration system (CC‐105; Tomy Seiko Co., Tokyo, Japan). The residue was dissolved in the HPLC mobile phase and injected into an LC‐MS/MS system. For quantification of genistein and GEN7G in the culture fluids, a portion of them was diluted with water and mixed with internal standard solution and ethyl acetate, and the solution was centrifuged at 7000*g* for 5 min. The supernatant was dried and concentrated under a stream of nitrogen gas. The residue was diluted with the HPLC mobile phase and injected into the LC‐MS/MS system.

HPLC separation of genistein and GEN7G was performed using an Agilent 1100 system (Agilent Technologies, Santa Clara, CA), which consisted of a vacuum degasser and a quaternary pump for solvent and sample delivery. A Kinetex PFP column (100 × 2.1 mm I.D., 2.6‐µm particle size; Phenomenex, Torrance, CA) was used to separate each analyte for LC‐MS/MS at 40°C. The mobile phase consisted of solution A (0.2 vol% acetic acid) and solution B (acetonitrile containing 0.2 vol% acetic acid) with a gradient of solution B (0–10 min, 11%; 10–30 min, 11–40%; 30–30.01 min, 40–90%; 30.01–35 min, 90%; 35–35.01 min, 90–11%; 35.01–40 min, 11%; v/v) at a flow rate of 0.35 mL/min. The HPLC system was interfaced with an API4000 triple quadrupole mass spectrometer (AB Sciex, Framingham, MA). The mass spectrometric analyses were conducted using electrospray ionization operating in negative ionization mode using the following multiple reaction monitoring (MRM) mass transitions (*m/z*): 268.919/132.7 (genistein) and 445.072/269.1 (GEN7G). The declustering potential was set at −105 or −85 V, the collision energy was set at −42 or −30 V, and collision cell exit potential was set at −21 or −19 V for analyses of genistein and GEN7G, respectively. Other parameters were set as follows: curtain gas, 40; ion‐spray voltage, −4500 V; temperature, 600°C; ion source gas 1, 50 psi; ion source gas 2, 40 psi; and collision‐activated dissociation gas, 6 psi.

### Evaluation of phagocytosis activity in pseudoinfection model

The phagocytosis of immune cells in the inflamed ears was evaluated according to the report published previously with a minor modification 15. GEN7G was injected intravenously into mice at 1 mg/kg in saline immediately and 3 h after the bacterial injection. The ears were dissected 6 h post and cut into nine pieces, followed by enzymatic digestion in PBS containing 0.45 mg/mL dispase I (Rosche Diagnostics, Indianapolis, IN), 2 mg/mL collagenase II (Sigma–Aldrich), and 0.32 mg/mL DNase I (Sigma–Aldrich) for 2 h while shaking at 37°C. Single‐cell suspensions were prepared by mechanical disruption in ice‐cold DMEM supplemented with 10% FBS, followed by filtration through a 70‐µm cell strainer. After centrifugation twice, cells were gently suspended in 30% Percoll (GE Healthcare, Piscataway, NJ), and then centrifuged at 630*g* for 20 min to isolate and remove sebaceous, low‐density cells, and debris. Cell pellets were resuspended in DMEM and passed through a 40‐µm cell strainer. After centrifugation, cells were fixed with paraformaldehyde and stored at 4°C until flow cytometric analysis. After blocking non‐specific binding using mouse IgG1 (clone: MG1‐45, BioLegend, San Diego, CA) and mouse FcBlocker (BD Bioscience, San Diego, CA), the following antibodies were reacted: PE anti‐mouse CD45 (clone: 30‐F11, BD Bioscience), PE/Cy7 anti‐CD11b (clone: 30‐F11, BioLegend), and biotin anti‐Ly6G (clone: 1A8, BioLegend). APC‐streptavidin (BioLegend) was used as the secondary reagent. Cell samples were analyzed with a FACSaria II flow cytometer and DIVA 8.0.1 software (BD Biosciences). Debris (FCS vs. SSC) and doublets (FSC‐H vs. FSC‐A) were excluded. Cells from the ears of naïve mice showed cell populations that were approximately 15% CD45‐positive and approximately 8% CD11b‐positive in the whole cells. CD11b^+^Ly6G^−^ and CD11b^+^Ly6G^+^ cells were regarded as monocytes/macrophages and neutrophils, respectively.

### Macrophage assays

Mouse macrophage RAW264.7 cells (ATCC, Manassas, VA) were grown in DMEM supplemented with 10% heat‐inactivated fetal bovine serum, 4.5 g/L glucose, 2 mmol/L l‐glutamine, 100 U/mL penicillin, 100 μg/mL streptomycin, and 10 mmol/L HEPES. Cells were seeded in 96‐well culture plates at 5–20 × 10^3^ cells/well, and cultured with the test compound (1–30 µmol/L) in the presence or absence of a suboptimal dose of 0.5 ng/mL mouse IFN‐γ (PeproTech, Rocky Hill, NJ). One to three days after incubation in a 5% CO_2_‐gas incubator, cells were harvested and analyzed by phagocytosis or cell‐surface antigen expression assay.

To evaluate phagocytic activity, FITC‐*S. aureus* prepared in the medium described above was added at a final concentration of 30 µg/mL after removal of culture fluids. After 30 min incubation in a 5% CO_2_‐gas incubator, cells were harvested using cold PBS containing 2 mmol/L EDTA, washed, and treated for 15 min with phosphate buffer containing 4% paraformaldehyde (pH 7.4). FITC‐positive cells were quantified by FACS. Activity was indicated as mean fluorescent intensity (MFI) of whole cells. Some tests were done in the presence of the β‐glucuronidase inhibitor 1‐((6,8‐dimethyl‐2‐oxo‐1,2‐dihydroquinolin‐3‐yl)methyl)‐3‐(4‐ethoxyphenyl)‐1‐(2‐hydroxyethyl) thiourea (Calbiochem, San Diego, CA) or estrogen‐receptor antagonist ICI‐182780 (Sigma–Aldrich).

In assays to measure cell‐surface antigen expression, the harvested cells were treated with anti‐CD16/32 antibody, followed by incubation on ice for 20 min with FITC‐labeled anti‐mouse CD86 (clone: GL1) purchased from BD Biosciences, APC‐labeled anti‐mouse CD192 (clone: SA203G11), PE/Cy7‐labeled anti‐mouse CD11b (clone: M1/70), or CD88 (clone: 20/70), which were all purchased from BioLegend. After washing, cells were treated for 15 min with phosphate buffer containing 4% paraformaldehyde, and analyzed using the FACS system. Levels of expression of cell‐surface antigen were all indicated as MFI. Fluorescent‐labeled isotype‐matched control antibodies (BD Biosciences and BioLegend) were used in this study, confirming that the antibodies showed specific binding.

### Assays of interaction with estrogen receptors

Binding assays from Eurofins Panlabs Taiwan Ltd. (Taipei, Taiwan) were performed targeting human nuclear estrogen receptors. Human estrogen receptor‐α (ERα) and estrogen receptor‐β (ERβ) expressed in Sf9 insect cells were prepared individually in modified Tris–HCl buffer pH 7.4. An aliquot of 9.6 ng (ERα) or 7.5 ng (ERβ) was incubated with 0.5 nmol/L [^3^H]‐estradiol for 2 h at 25°C. Non‐specific binding was estimated in the presence of 1 µmol/L diethylstilbestrol. Receptor proteins were filtered and washed, and the filters were then counted to determine specifically bound [^3^H]‐estradiol. The specific binding was defined by subtracting nonspecific binding from total binding, and expressed as the percentage inhibition using the following formula: inhibition (%) = (1−[c−a]/[b−a]) × 100, where “a” is the average cpm of nonspecific binding, “b” is the average cpm of total binding, and “c” is the average cpm in the presence of the test substance. The binding specificities of these procedures were approximately 85% (ERα) and 90% (ERβ). The half maximal inhibitory concentration (IC_50_) of test samples was calculated.

A PathHunter β‐arrestin assay provided by DiscoveRx Corp. (Fremont, CA) was performed using CHO‐K1 cells engineered to coexpress ProLink‐tagged GPR30, which is called human G‐coupled estrogen receptor 1, and β‐galactosidase acceptor‐tagged β‐arrestin. The cells were seeded in 384‐well microplates and incubated with test samples for 90–180 min, followed by incubation with the PathHunter detection reagent cocktail for 1 h. Chemiluminescent signals were measured using a PerkinElmer EnVision reader, and evaluated using the CBIS data analysis suite (ChemInnovation Software Inc., San Diego, CA). The percentage activity was calculated using the following formula: activity (%) =100 × (mean relative luminescent units (RLU) of test sample—mean RLU of vehicle control)/(mean RLU of vehicle control). Mean RLU of positive and negative controls were 2,178,667 and 648,267, respectively.

### Measurement of concentration and enzymatic activity of β‐glucuronidase

Total RNA was prepared from proliferating RAW264.7 cells cultured in 24‐well plates, followed by preparation of cDNAs. A TaqMan gene expression assay was performed using TaqMan primers for Gusb (β‐glucuronidase) and a housekeeping gene, Gapdh (glyceraldehyde‐3‐phosphate dehydrogenase), which were purchased from ABI Biosystems (Foster City, CA). To measure the amount of β‐glucuronidase in a level of protein, culture fluids and lysates of RAW264.7 cells and homogenizations of the mouse skins were centrifuged at 10,000*g* for 20 min at 4°C, and stored at −80°C. β‐glucuronidase was quantified using ELISA kits according to the manufacturer's instructions (Cloud‐Clone Corp., Houston, TX). Total protein was measured by the Bradford assay (Bio‐Rad, Hercules, CA) using bovine serum albumin as a standard. Determinations of β‐glucuronidase were compensated and are indicated as relative amounts.

To examine β‐glucuronidase activity, GEN7G was added as a substrate at a final concentration of 30 µmol/L to live RAW264.7 cells seeded at 1 × 10^5^ cells/0.5 mL/well in 24‐well plates, or its lysates. After 24 h incubation in a 5% CO_2_‐gas incubator, the culture fluids or lysates were harvested and stored at −80°C until measurement of genistein and GEN7G by LC‐MS/MS as described above.

### Statistical analysis

All values are expressed as the mean ± SEM. Statistical significance was evaluated by one‐way analysis of variance (ANOVA) or two‐way repeated measures ANOVA, followed by Dunnett's multiple comparisons or unpaired Student's *t*‐test. A probability of less than 0.05 was considered significant.

## Results

### Flavonoid glucuronide GEN7G is converted to aglycone genistein in inflamed mouse skin

**Figure 1:**
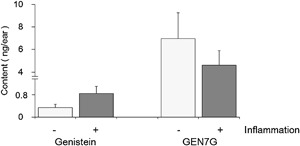
Conversion of intravenously administered genistein 7‐*O*‐glucuronide to aglycone in the skin. Particles of FITC‐conjugated and killed *S. aureus* (67 µg/10 µl/site) were injected intradermally into the one side of the ears of mice. Genistein 7‐*O*‐glucuronide (GEN7G) dissolved in saline was administered intravenously to the mice at a dose of 1 mg/10 mL/kg immediately and 3 h after the pseudoinfection. The inflamed and non‐inflamed ears were cut off at 6 h after the pseudoinfection, and stored at −80°C until use. The ears were homogenized in PBS containing 20 mmol/L saccharic acid 1,4‐lactone, followed by measurement of GEN7G and genistein by LC‐MS/MS. Data are shown as mean ± SEM of triplicate tests.

It is well demonstrated that oral administration of genistein exerts a variety of biological effects and that genistein is conjugated predominantly to GEN7G in the gut, as reported elsewhere 16. Using a mouse model of skin inflammation in which FITC‐labeled *S. aureus* was injected intradermally into the ears, we first examined whether genistein was detectable in the inflamed skin after intravenous injection of GEN7G. Both genistein and GEN7G were detected in both bacteria‐injected inflamed skin and non‐injected normal skin (Fig. 1). The amount of genistein was 2.5 times higher in the inflamed skin (0.843 ng/ear) than in the non‐injected skin (0.338 ng/ear). On the other hand, the concentration of GEN7G was slightly lower in inflamed skin than in non‐inflamed skin, though the difference was not statistically significant. No genistein was found in the skin of normal mice without genistein injection (data not shown).

We evaluated the effect of GEN7G on phagocytosis using immune cells in vivo. To quantitate numbers, populations, and phagocytic activity of monocytes/macrophages (CD11b^+^Ly6G^−^) and neutrophils (CD11b^+^Ly6G^+^) in the inflamed ears, FACS analysis was performed at 6 h post pseudoinfection in mice injected with GEN7G intravenously. The bacteria inoculation (Control and GEN7G) did not alter the total number of CD11b^+^Ly6G^−^ cells compared to normal skin (normal). The MFI of FITC in FITC‐positive CD11b^+^Ly6G^−^ cells, which represent the monocytes/macrophages ingesting FITC‐labeled bacteria, were significantly higher in GEN7G‐treated group than in the Control (Table 1 and representative blots are shown in Supplemental Fig. S2). In contrast, the MFI of FITC in CD11b^+^Ly6G^+^ cells (neutrophils) were not significantly different between bacteria‐injected control and GEN7G‐treated groups, though the cell numbers and phagocytic activity of CD11b^+^Ly6G^+^ cells were clearly increased in the pseudoinfection model. These findings suggest an adjuvant effect of GEN7G on enhancing phagocytic response of macrophages in vivo.

**Table 1:** Cytometric analysis of phagocytes in ears of mice treated with genistein 7‐*O*‐glucuronide

		Cell number (×10^4^ cells per ear)			MFI of FITC	
	Region	Normal	Control	GEN7G	Control	GEN7G
Monocytes/macrophages						
CD11b^+^ Ly6G^−^ (whole)	R1	31.9 ± 0.6	33.3 ± 1.8	31.6 ± 2.0	50 ± 6	65 ± 11
CD11b^+^ Ly6G^−^ FITC^+^	R3	–	4.5 ± 0.4	3.9 ± 0.7	255 ± 14	404 ± 54#
Neutrophils						
CD11b^+^ Ly6G^+^ (whole)	R2	0.4 ±	35.1 ± 4.9	35.4 ± 7.3	1994 ± 235	2537 ± 479
CD11b^+^ Ly6G^+^ FITC^+^	R4	–	23.0 ± 4.2	24.2 ± 5.5	3108 ± 300	3591 ± 494

Particles of FITC‐conjugated and killed *S. aureus* (67 µg/10 µl/site) were injected intradermally into the ears of mice. Genistein 7‐*O*‐glucuronide (GEN7G) dissolved in saline was administrated intravenously to the mice at a dose of 1 mg/10 ml/kg immediately and 3 h post pseudoinfection. The ears were cut off 6 h post pseudoinfection, and digested in a mixture of three enzymes (dispase I, collagenase II, and DNase I) for 2 h at 37°C, followed by preparation of single cells. Phagocytic cells were stained by anti‐mouse Ly6G and anti‐mouse CD11b, and analyzed by flow cytometry using an FACSaria II system. Cell numbers and mean fluorescent intensity (MFI) of FITC in neutrophils (CD11b^+^Ly6G^+^) and monocytes/macrophages (CD11b^+^Ly6G^−^) are shown as mean ± SEM of 4 (Normal), 8 (Control and GEN7G) ears per group. Total numbers of harvested ear cells were 422 ± 10, 433 ± 17, and 404 ± 16 × 10^4^, respectively, showing no difference among groups. R1, R2, R3, and R4 indicate each region built in dot plots of Supplemental Figure S2. #*P* < 0.05 significance versus control group at Student *t*‐test.

### Flavonoid glucuronides GEN7G and LQG7G enhanced macrophage phagocytosis and functional receptor expression

**Figure 2:**
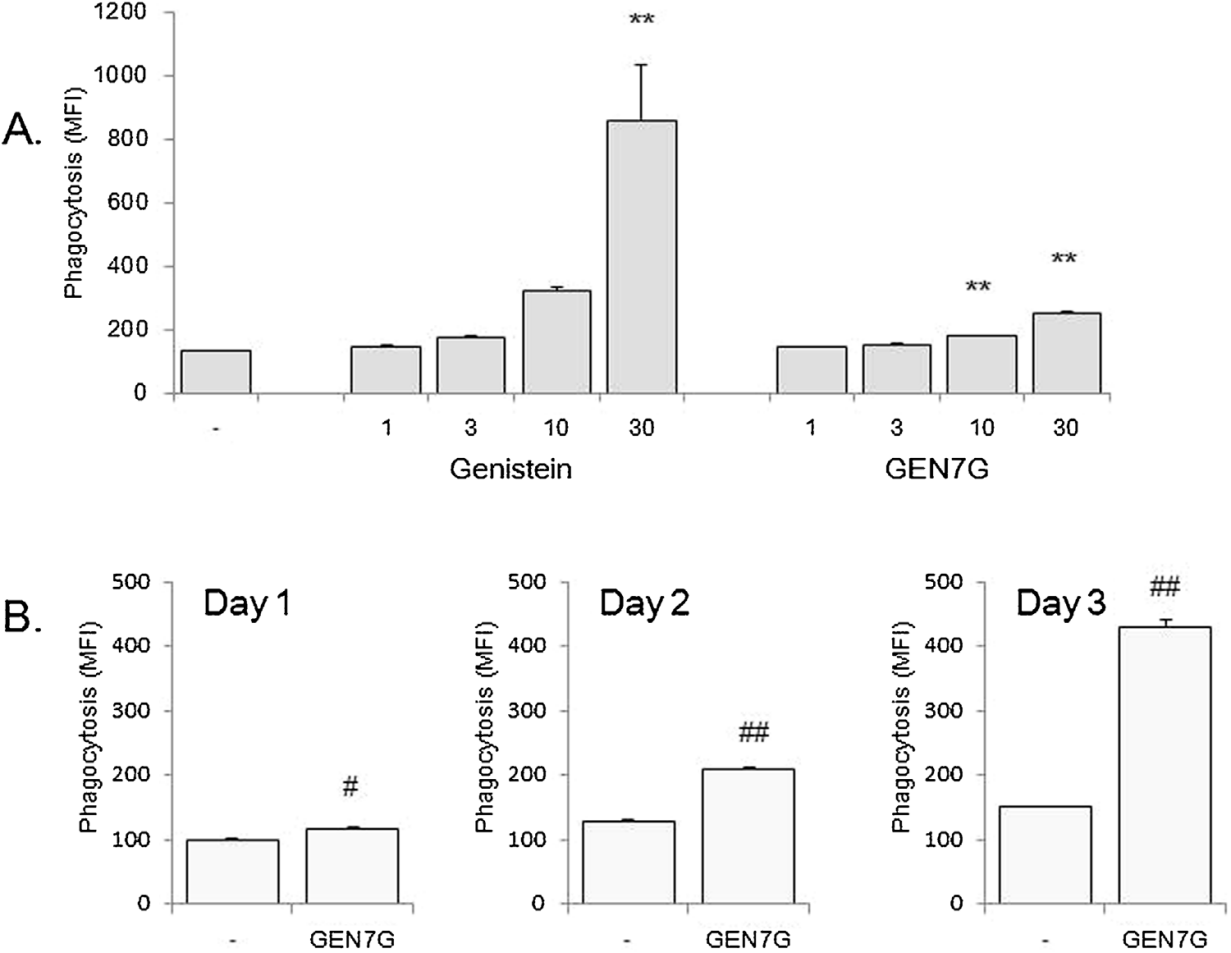
Dose‐ and time‐dependency of genistein 7‐*O*‐glucuronide on macrophage phagocytosis. RAW264.7 cells were cultured for 1, 2, and 3 days in the presence of a suboptimal dose of IFN‐γ (0.5 ng/mL), followed by examination of phagocytic activity. Dose‐dependency (A) and time‐dependency (B) of genistein 7‐*O*‐glucuronide (GEN7G) and/or genistein were examined. Data are shown as mean ± SEM of triplicate tests. **P < 0.01 significance versus vehicle control by Dunnett's test. #, ##*P* < 0.05, 0.01 significance versus vehicle control by Student's *t*‐test, respectively.

**Figure 3:**
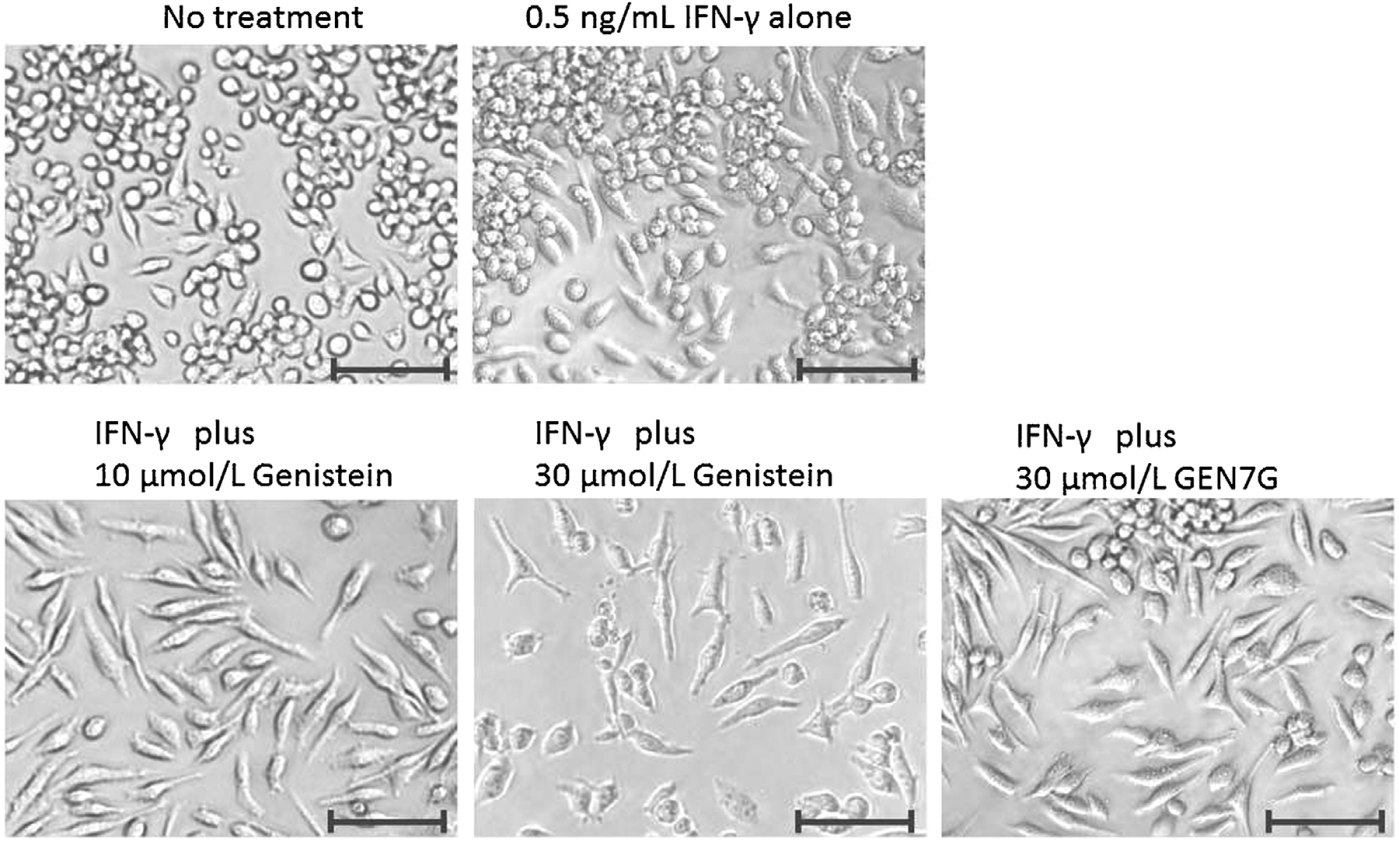
Morphological changes in RAW264.7 cells due to genistein and genistein 7‐*O*‐glucuronide. RAW264.7 cells were cultured for 3 days in the presence of a suboptimal dose of IFN‐γ (0.5 ng/mL) plus the indicated test sample. Bars: 50 µm.

To investigate the bioactive characteristics of GEN7G in the macrophage culture systems, we examined and compared various types of flavonoid glucuronides that had been quantified in the plasma of rats given orally with JHT^13^, as well as their aglycones, genistein, liquiritigenin, and hesperetin. As shown in Table 2, GEN7G and LQG7G as well as their aglycones significantly enhanced phagocytosis in RAW264.7 cells in the presence of a suboptimal dose of IFN‐γ (0.5 ng/mL), while LQG4′G, HPT7G, and hesperetin did not. GEN7G, which showed the highest activity among the four tested flavonoid‐glucuronides, enhanced the phagocytic activity of RAW264.7 cells in a dose‐ and time‐dependent manner up to three days (Fig. 2A and B). We also observed that addition of GEN7G and its aglycone genistein turned cell morphology of RAW264.7 cells to be more adhesive and spindle shaped in the presence of IFN‐γ (Fig. 3).

**Table 2:** Phagocytosis assay by flavonoid glucuronides and aglycones

Test sample	Phagocytosis (MFI)
Glucuronide	
Vehicle control	149 ± 3
Genistein 7‐*O*‐glucuronide	247 ± 4**
Liquiritigenin 7‐*O*‐glucuronide	180 ± 3**
Liquiritigenin 4’‐*O*‐glucuronide	138 ± 2
Hesperetin 7‐*O*‐glucuronide	146 ± 7
Aglycone	
Vehicle control	143 ± 2
Genistein	876 ± 33**
Liquiritigenin	280 ± 11**
Hesperetin	139 ± 5

RAW264.7 cells were cultured for 3 days with 30 µmol/L of flavonoid glucuronide or the aglycone in the presence of a suboptimal dose of IFN‐γ (0.5 ng/ml). Phagocytic activity was examined using FITC‐conjugated and killed *S. aureus*, representing mean fluorescent intensity (MFI). Data are shown as mean ± SEM of triplicate tests. **P < 0.01 significance versus vehicle control by Dunnett test.

We next performed flow cytometric analysis of the following functional receptors on RAW264.7 cells: chemotactic receptor CD192 for monocyte chemoattractant protein‐1 (MCP‐1), complement receptors CD88, and CD11b for components C5a and iC3b, respectively, and costimulatory molecule CD86. Three‐day culture in the presence of GEN7G or LQG7G increased their expression, while LQG4′G and HPT7G were inactive (Table 3). Again, parallel to the GEN7G and LQG7G effects on RAW264.7 cells, their aglycone forms genistein and liquiritigenin but not hesperetin increased the functional antigens.

**Table 3:** Flow cytometric analysis to examine effect of flavonoids on functional surface antigen expression

	Expression of surface antigen (MFI)			
Test sample	CD11b	CD86	CD88	CD192
Vehicle control	544 ± 23	542 ± 27	447 ± 20	126 ± 9
Genistein 7‐*O*‐glucuronide	882 ± 4**	961 ± 77**	1082 ± 18**	247 ± 26**
Liquiritigenin 7‐*O*‐glucuronide	621 ± 15	485 ± 64	653 ± 23**	204 ± 7**
Liquiritigenin 4’‐*O*‐glucuronide	551 ± 24	498 ± 49	514 ± 24	133 ± 8
Hesperetin 7‐*O*‐glucuronide	537 ± 24	546 ± 79	485 ± 27	144 ± 7
Genistein	931 ± 44**	2587 ± 127**	844 ± 38**	169 ± 12
Liquiritigenin	628 ± 13	529 ± 3	805 ± 42**	262 ± 14**
Hesperetin	455 ± 19	643 ± 7	467 ± 23	96 ± 9

RAW264.7 cells were cultured for 3 days with 30 µmol/L of flavonoid glucuronide in the presence of a suboptimal dose of IFN‐γ (0.5 ng/ml). Surface antigens, CD11b, CD86, CD88, and CD192 were analyzed using their specific antibodies, representing mean fluorescent intensity (MFI) subtracted from the respective MFI of the isotype control antibody. Expressions of cells in no IFN‐γ wells were 621 ± 12 (CD11b), 378 ± 74 (CD86), 393 ± 1 (CD88), and 14 ± 2 (CD192). Data are shown as mean ± SEM of triplicate tests. **P < 0.01 significance versus vehicle control by Dunnett test.

### Flavonoid‐glucuronide GEN7G enhanced macrophage phagocytosis in a nuclear estrogen receptor‐dependent manner

**Figure 4:**
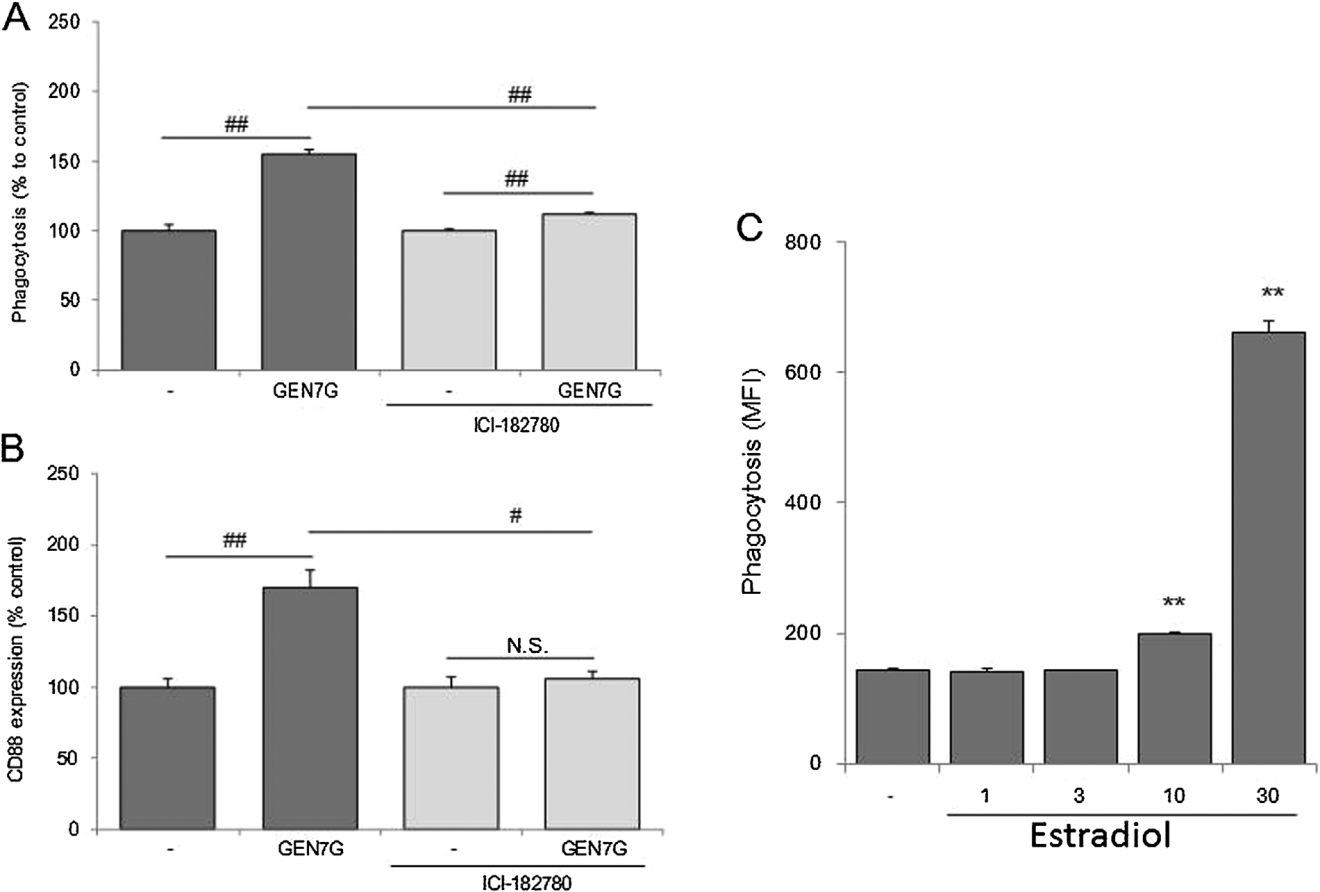
Involvement of signaling via nuclear estrogen receptor on macrophage activation of genistein 7‐*O*‐glucuronide. RAW264.7 cells were cultured for 3 days in the presence of a suboptimal dose of IFN‐γ (0.5 ng/mL) with genistein 7‐*O*‐glucuronide (GEN7G, 30 µmol/L), estradiol (1, 3, 10, 30 µmol/L), or vehicle. The estrogen receptor antagonist ICI‐182780 was added at 5 µmol/L, three times on days 0, 1, and 2. Phagocytosis (A and C) and CD88 expression (B) were analyzed, showing data as mean ± SEM of triplicate tests. The reproducibility was confirmed in several separate examinations. **P < 0.01 significance versus vehicle control by Dunnett's test, #, ##*P* < 0.05, 0.01 significance by Student's *t*‐test, respectively.

Because genistein and liquiritigenin are reported to bind to nuclear estrogen receptors (ERs) 17, 18, we investigated the involvement of nuclear ERs in flavonoid effects on macrophages. Addition of the nuclear ER antagonist ICI‐182780 significantly suppressed phagocytosis and abolished the increase in CD88 expression induced by GEN7G (Fig. 4A and B). In contrast, the nuclear ER agonist estradiol enhanced phagocytosis at concentrations similar to those of GEN7G and genistein (Fig. 4C).

### Flavonoid aglycones genistein and liquiritigenin but not glucuronides GEN7G and LQG7G directly bind to nuclear estrogen receptors

**Figure 5:**
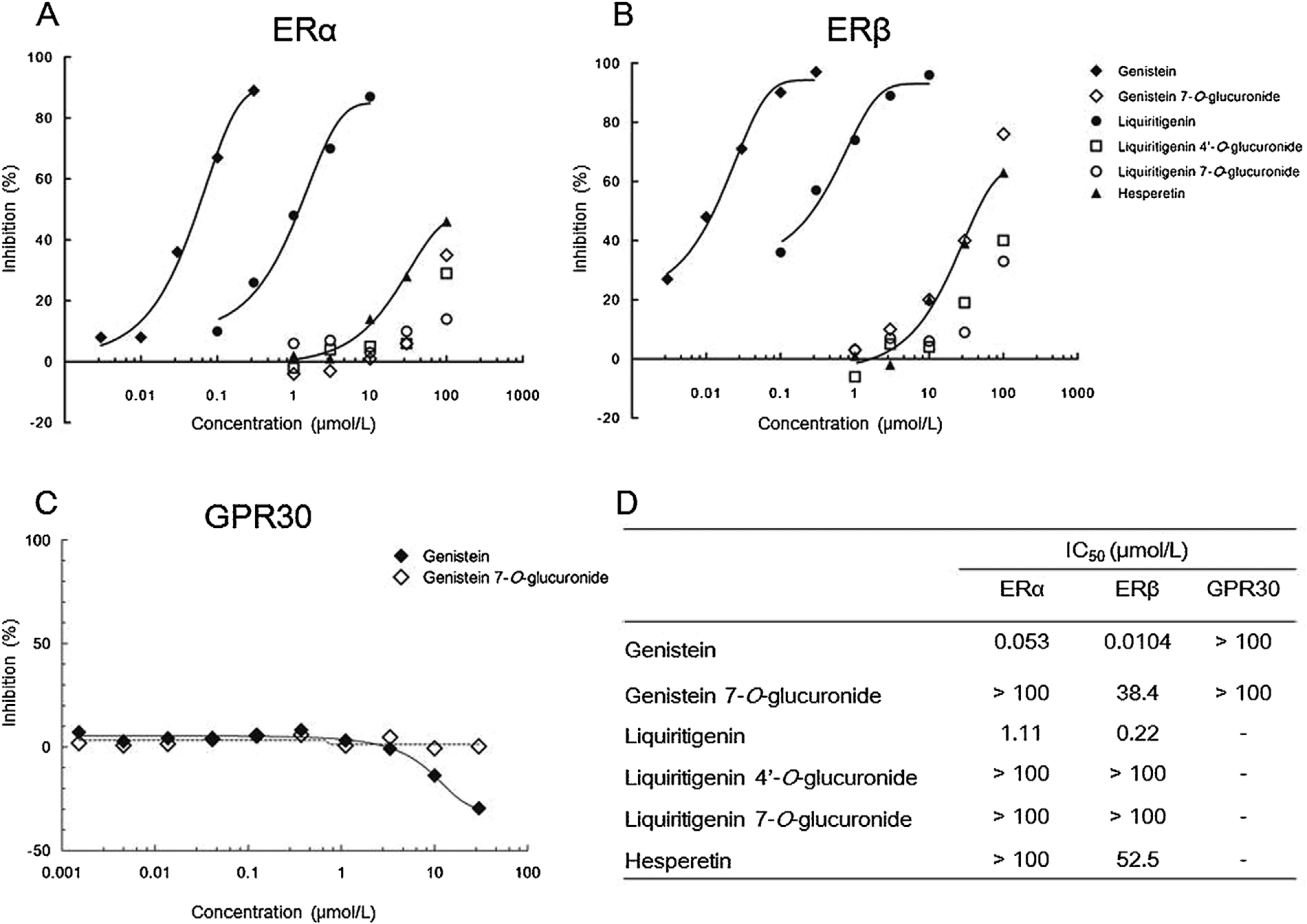
Molecular‐based interaction of flavonoids to estrogen receptors. (A and B) Aliquots of human estrogen receptor‐α (ERα) and estrogen receptor‐β (ERβ) were incubated for 2 h with 0.5 nmol/L [^3^H]‐estradiol in the presence of the test flavonoid at the indicated concentrations. Receptor proteins were filtered and washed, the filters are then counted to determine specifically bound [^3^H]‐estradiol. The concentration‐dependent curves are shown for ERα (A) and ERβ (B). (C) A β‐arrestin assay was performed using CHO‐K1 cell engineered to co‐express ProLink‐tagged GPR30 (human G‐coupled estrogen receptor 1), and β‐galactosidase acceptor‐tagged β‐arrestin. The cells were seeded in 384‐well microplates and incubated with the test flavonoid at the indicated concentrations, followed by incubation with a detection reagent cocktail for 1 h. Chemiluminescent signals were measured and are shown as the percentage activity. The concentration‐dependent curves are shown in GPR30. (D) A list of the half maximal inhibitory concentration (IC_50_) is shown. *N* = 2 in all assays.

Competitive binding assays were performed to examine direct binding of flavonoid glucuronides and aglycones to human ERs. The IC_50_s of genistein, liquiritigenin, and hesperetin were 0.053, 1.11, and >100 µmol/L in the ERα binding assay, and 0.010, 0.22, and 52.5 µmol/L in the ERβ binding assay, respectively (Fig. 5A, B, and D). On the other hand, the IC_50_s of GEN7G, LQG7G, and LQG4′G were more than 30 µmol/L in both ERα and ERβ binding assays. We further examined the interaction of flavonoids with membrane‐associated GPR30, one of the G‐protein‐coupled estrogen receptor (GPERs). As shown in Fig. 5C and D, genistein did not exhibit agonistic activity toward GPR30 as shown by a reduction of the basal signal following β‐arrestin recruitment. GEN7G also did not exert a reaction against human GPR30 even at the high concentration of 100 µmol/L. These findings suggested that the aglycones genistein and liquiritigenin, but not the glucuronides GEN7G and LQG7G, could directly bind to ERα and ERβ but not to membrane‐associated GPR30.

### Macrophages express β‐glucuronidase and convert flavonoid glucuronides GEN7G to aglycones genistein

**Figure 6:**
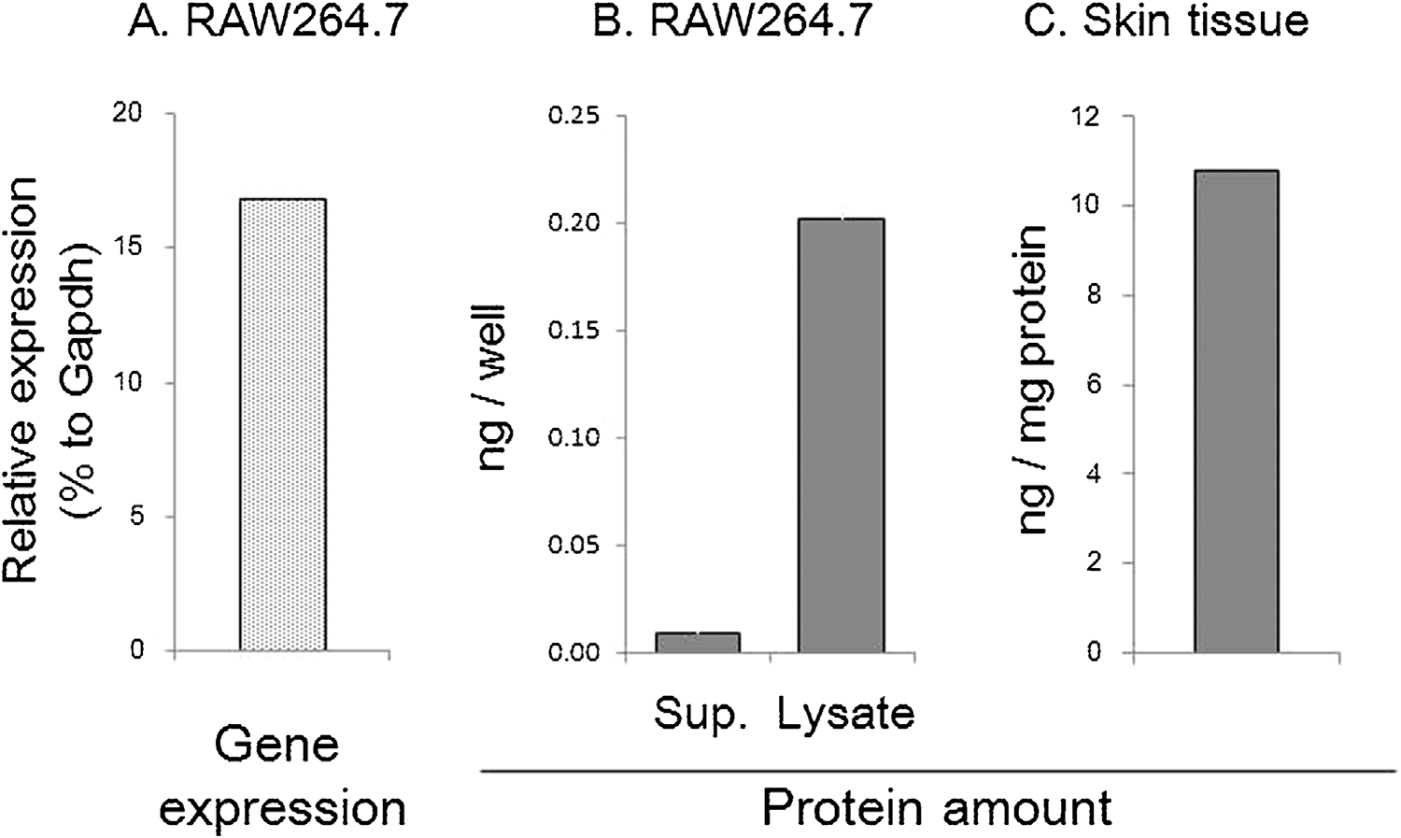
Measurement of β‐glucuronidase in macrophage sources. (A) Total RNAs were prepared from proliferating RAW264.7 cells, followed by preparation of cDNAs. TaqMan gene expression assays were performed using TaqMan primers for Gusb (β‐glucuronidase) and Gapdh (glyceraldehyde‐3‐phosphate dehydrogenase). *N* = 2. (B) RAW264.7 cells cultured overnight was cultured for an additional 24 h after replacing the culture fluid with fresh medium. The culture supernatants and 0.1% Triton X‐treated cellular lysates were harvested. The amounts of β‐glucuronidase per well were measured by ELISA method. *N* = 3, (C) normal ears were cut off, homogenized, and β‐glucuronidase per mg protein was measured. *N* = 1.

**Figure 7:**
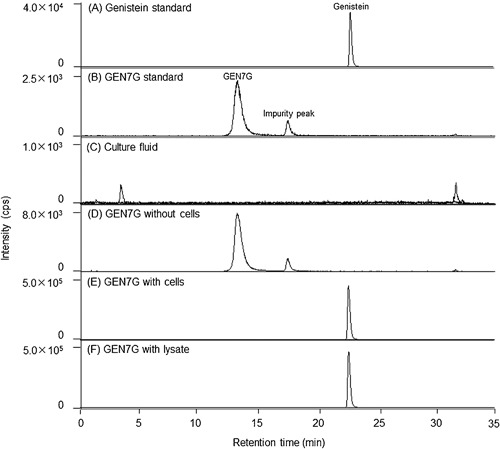
Deconjugation of genistein 7‐*O*‐glucuronide to aglycone in RAW264.7 cells. Multiple reaction monitoring (MRM) chromatograms of genistein standard (A), genistein 7‐*O*‐glucuronide (GEN7G) standard (B), the supernatants incubated for 24 h in 24‐well plates under the following conditions: live RAW264.7 cells alone seeded at 1 × 10^5^ cells/0.5 mL/well (C), 30 µmol/L GEN7G alone (D), 30 µmol/L GEN7G, and the live cells (E), 30 µmol/L GEN7G incubated for 24 h with the cell lysate prepared by sonicating RAW264.7 cells seeded at 1 × 10^5^ cells/well (F).

**Figure 8:**
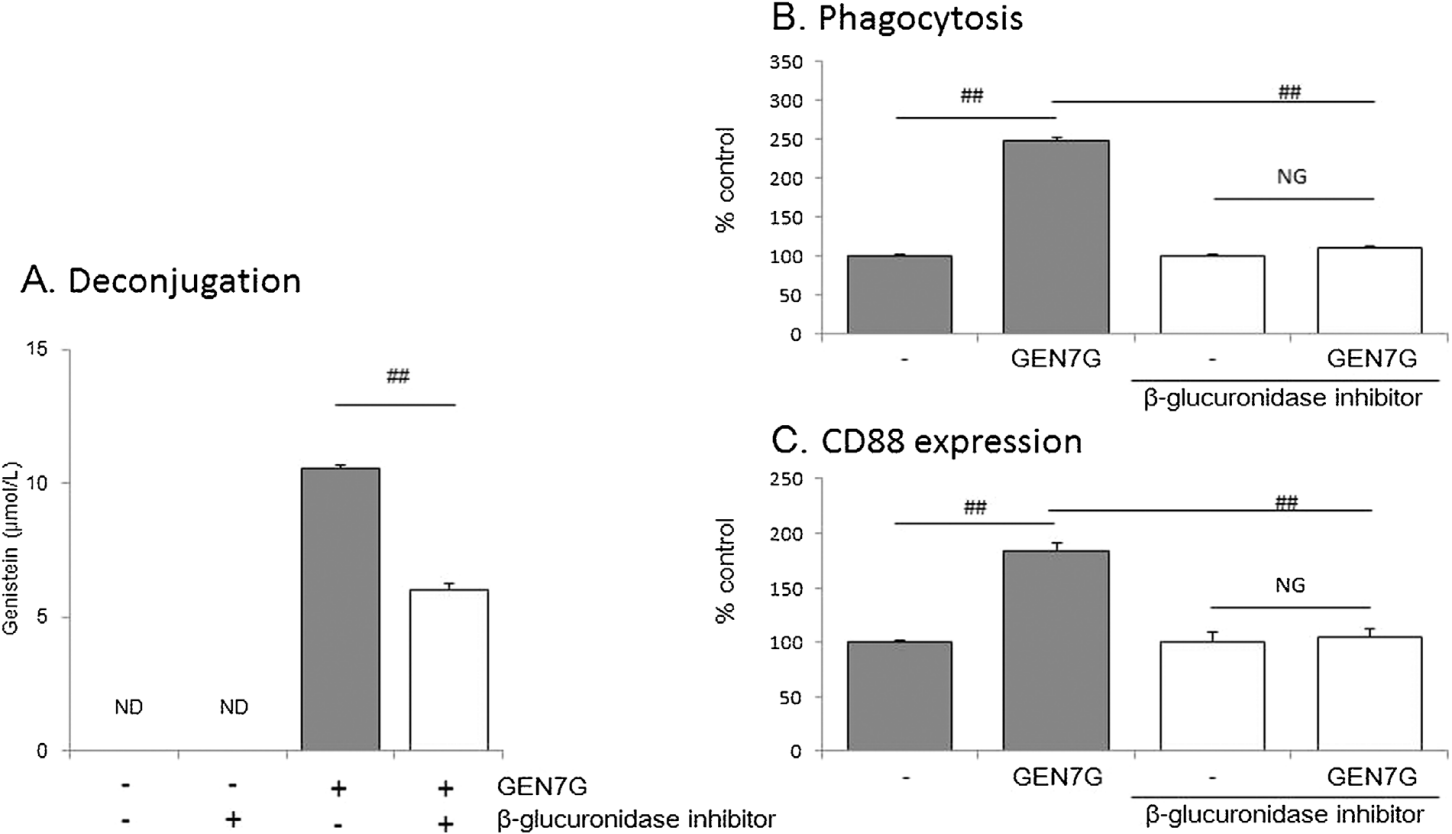
Involvement of deconjugation process in macrophage activation of genistein 7‐*O*‐glucuronide. RAW264.7 cells were cultured for 3 days in the presence of a suboptimal dose of IFN‐γ (0.5 ng/mL) with or without 30 µmol/L genistein 7‐*O*‐glucuronide (GEN7G). A β‐glucuronidase inhibitor 1‐((6,8‐dimethyl‐2‐oxo‐1,2‐dihydroquinolin‐3‐yl)methyl)‐3‐(4‐ethoxyphenyl)‐1‐(2‐hydroxyethyl) thiourea was added at 10 µmol/L every day. The deconjugated genistein (A) in the culture fluids was measured by LS/MS/MS, and phagocytosis (B) and CD88 expression (C) of cells were evaluated. Data are shown as mean ± SEM of Ta tests. The reproducibility was confirmed in several separate examinations. ND: Not detected, ##*P* < 0.01 significance by Student's *t*‐test.

These lines led us to hypothesize that a deconjugation process of the glucuronides GEN7G and LQG7G to the aglycones genistein and liquiritigenin, respectively, is required for flavonoids to enhance macrophage phagocytosis. We examined and confirmed that RAW264.7 cells expressed β‐glucuronidase at the levels of messenger RNA and protein (Fig. 6A and B). We also detected β‐glucuronidase in mouse whole skin (Fig. 6B). We next examined whether β‐glucuronidase in RAW264.7 cells can deconjugate GEN7G to genistein. As shown in Figure 7, incubation of GEN7G in the presence of live cells or homogenized cell lysates converted GEN7G to genistein. The skin homogenate also showed a similar conversion of GEN7G to genistein (data not shown). To verify the involvement of the deconjugation process in the macrophage activation induced by GEN7G, a β‐glucuronidase inhibitor was added to the cultures. The conversion to genistein in RAW264.7 cells was reduced to half by the β‐glucuronidase inhibitor (Fig. 8A). The β‐glucuronidase inhibitor completely abolished the increase in phagocytosis and CD88 expression of RAW264.7 cells induced by GEN7G (Fig. 8B and C).

## Discussion

It was revealed for the first time that GEN7G enhanced phagocytosis and expressions of functional surface antigens in macrophages, and that intravenous administration of GEN7G enhanced phagocytosis of CD11b^+^Ly6G^−^ cells in the inflamed ears. The most important finding of the present study was confirmation of the consistent effects of GEN7G in vitro and in vivo because glucuronides have been generally recognized to be inactive. Our finding has raised the necessity of further study focusing on glucuronides as a carrier and precursor form of flavonoids.

**Figure 9:**
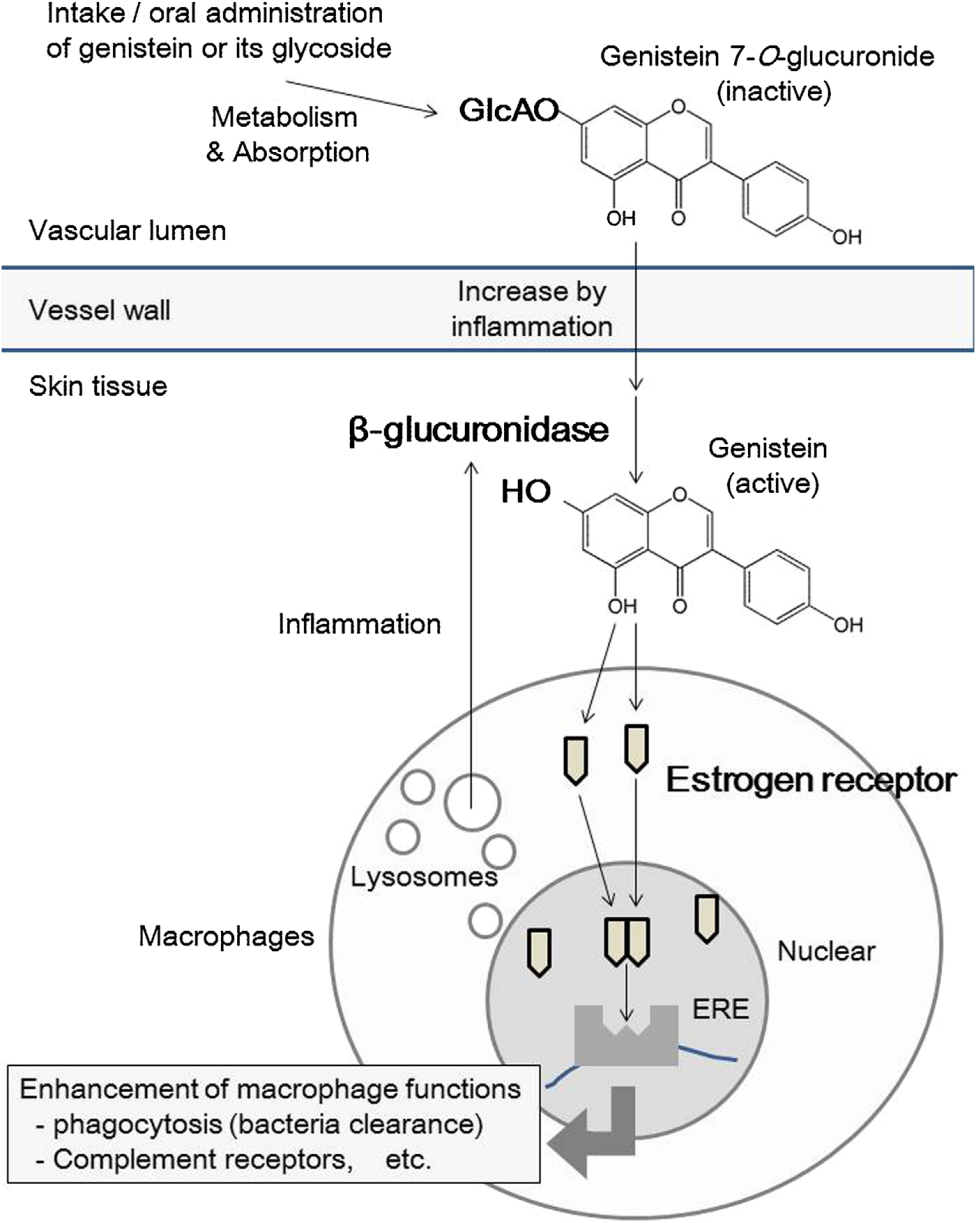
Possible mechanisms of macrophage activation by genistein 7‐*O*‐glucuronide. (1) GEN7G infiltrates to the inflamed sites through increased microvascular permeability; (2) GEN7G is exposed to resident macrophages and converted to genistein by β‐glucuronidase of resident macrophages intra‐ and extracellularly; (3) thereafter, resident macrophages are more easily activated by genistein and GEN7G than neutrophils that accumulate later in the inflamed sites.

We concluded that the effects of GEN7G on macrophage functions depend on signaling via nuclear ERs, because (1) the enhancing activities of genistein, liquiritigenin, and hesperetin on macrophage phagocytosis were correlated to the binding activities to nuclear ERs 17; (2) the action of GEN7G was decreased by ICI‐182780, which works as an antagonist of nuclear ERs and also as an agonist of membrane GPERs 19; (3) estradiol enhanced macrophage phagocytosis at similar concentrations as genistein and GEN7G; (4) GEN7G showed no agonistic activity toward GPR30 even at the high concentration of 100 µmol/L, and genistein reduced intracellular signals following β‐arrestin recruitment, although some papers have demonstrated the agonism of genistein 20, 21; and (5) some flavonoids known as phytoestrogens enhance the phagocytic activity of myeloid leukemia cells and activate macrophage‐like cells 22, 23, 24, 25, 26, 27. Quantitative real‐time PCR revealed that RAW264.7 cells dominantly expressed Esr1/ERα, but not Esr2/ERβ (Supplemental Fig. S3). The signal pathway through nuclear ERs in immune cells, especially dendritic cells, and macrophages, was reviewed recently, and ERs, in particular ERα, may induce epigenetic changes in immune precursor cells that affect the downstream developmental pathway or functional responses in mature cells 28, 29, 30. Taken together, it is plausible to conclude that genistein/GEN7G promote macrophage function and differentiation through agonistic activity toward nuclear ERs and significantly involve innate immune systems (Fig. 9).

Contrary to the results of the present genistein/GEN7G study, several studies have demonstrated that various types of flavonoids inhibit macrophage activation 31, 32, 33, 34. Genistein also affects multiple receptor signaling. Genistein is used as a tyrosine‐kinase inhibitor and reported to reduce intracellular pro‐inflammatory signals such as NF‐κB 32, 35. Genistein is reported to be an agonist for nuclear receptors like PPAR‐γ and progesterone receptor as well as estrogen receptors 17, 36, 37. In our skin infection model, genistein/GEN7G enhanced CD11b^+^Ly6G^−^ tissue macrophages through ERs. Since genistein exerts profound effects and its function has multiple aspects in the inflammatory reaction, the precise analysis of macrophages types would be critical to understand the flavonoid action in inflammation.

Due to the sugar group, glucuronides are at a disadvantage in passing through the plasma membrane and interact with nuclear ERs localized in the cytoplasm and nucleus. Macrophage β‐glucuronidase is usually localized intracellularly in the lysosomes and appeared on cell surface, and released extracellularly by an increase of intracellular calcium ions, for example, LPS stimulation 32. In our culture systems, a suboptimal dose of IFN‐γ was added to increase the sensitivity to flavonoids, and we detected β‐glucuronidase in both supernatant and cell lysate of RAW264.7 cells. Therefore, the conversion of flavonoids can occur extracellularly, although intracellular conversion cannot be denied. The IC_50_ of GEN7G was approximately a 1000‐fold that of genistein in both ER1‐ and ER2‐binding assays. Addition of a β‐glucuronidase inhibitor completely suppressed the macrophage activation induced by GEN7G, though the conversion to genistein was reduced to half. As shown in Figure 2A, approximately 10 µmol/L of genistein was supposed to be the threshold concentration to activate macrophages. The β‐glucuronidase inhibitor reduced the genistein conversion to less than the speculated threshold concentration of ca. 7 µmol/L (Fig. 8A). Thus, the decrease in the genistein conversion to half might be enough to suppress macrophage activation completely by the β‐glucuronidase inhibitor. Another β‐glucuronidase inhibitor, saccharic acid 1,4‐lactone, also suppressed macrophage activation induced by GEN7G (data not shown). These lines of evidence indicate that a deconjugation process is a critical step to gain GEN7G activities.

The RAW264.7 cell is a line of immature macrophages. RAW264.7 cells showed a high potential to proliferate at least for one day after GEN7G stimulation, and cell proliferation gradually decreased after two to three days (data not shown). As shown in Figure 3, morphological changes of RAW264.7 cells were observed after two to three days stimulation with genistein and GEN7G. Phagocytosis of FITC‐conjugated bacteria was little at one‐day stimulation with GEN7G, and two to three days were required to observe clear evidence of enhanced phagocytosis as well as expression of functional receptors in vitro (Fig. 2 and Table 3). These observations suggest that genistein and GEN7G induce maturation of macrophages. On the other hand, phagocytosis of macrophages in the inflamed ears was significantly augmented at six hours after the first injection of GEN7G in vivo (Table 1). In living mice, mature and activated macrophages already existed in tissue as resident macrophages before flavonoid administration, and excessive maturation of macrophages may lead to pyroptosis, followed by release of intracellular bioactive factors to evoke a full‐fledged inflammatory response 38, 39. Given that dead cell‐derived extracellular β‐glucuronidase is sufficient for deconjugation of GEN7G or that macrophages highly possessing β‐glucuronidase are implicated in the inflamed sites, the appearance of GEN7G's effect would be observed quickly. The stages of macrophage maturation may also explain the time lag of enhanced phagocytosis by GEN7G between at two to three days in vitro and at six hours in vivo.

In the experiment shown in Figure 1, the amount of intravenously injected GEN7G was almost same between in inflamed skin and in non‐inflamed skin, or slightly lower in inflamed skin than in non‐inflamed skin. In contrast, the amount of the deconjugated genistein was 2.5 times higher in the inflamed skin than in the non‐injected skin. There is no doubt that GEN7G was metabolized to genistein in skin tissue, and the difference between GEN7G and genistein is supposed to result from inflammation‐induced conditions in which β‐glucuronidase works well. An increased vascular permeability is not enough to explain the difference.

The FACS analysis indicated enhancement of phagocytosis in FITC‐positive CD11b^+^Ly6G^−^ cells, which are monocytes/macrophages ingesting FITC‐conjugated bacteria. It was of interest that the activation by GEN7G was observed only in monocytes/macrophages but not in neutrophils. The increase of phagocytosis in monocytes/macrophages was speculated to depend on resident macrophages because monocytes are known to have little phagocytic activity 15. Along with stimulating the phagocytic function of resident macrophages, it is reported that β‐glucuronidase is expressed in resident macrophages but not circulating blood cells 32. Since we detected GEN7G after oral administration of JHT, we draw the dynamics of GEN7G as follows (Fig. 9): (1) GEN7G infiltrates inflamed sites through increased microvascular permeability; (2) GEN7G is exposed to resident macrophages and converted to genistein by the β‐glucuronidase of resident macrophages intra‐ and extracellularly; and (3) subsequently, resident macrophages are more easily activated by genistein and GEN7G than the neutrophils that accumulate later in the inflamed sites. In fact, genistein was found in the skin of mice given an intravenous injection of GEN7G. Generation of genistein by intravenous injection of GEN7G was higher in the inflamed ears than the non‐inflamed ears. The conversion of GEN7G to genistein was confirmed also by incubation with skin homogenizations of mouse ears (data not shown). These results raised a possibility that the diseased tissues or organs may be affected more effectively than normal tissue by flavonoid glucuronides, namely, orally administrated flavonoids. In general, aglycones are bioactive but unstable, while glucuronide conjugates are inactive but stable. Our data suggest that the macrophage‐derived deconjugation plays an important role in transiently providing bioactive/unstable aglycones in the biological fluids, in particular, within the sites of inflammation. The selective deconjugation within the sites of inflammation might ensure the safety of flavonoids in normal tissues.

From the standpoint of pharmacokinetic and metabolic studies, we clarified the mechanism by which orally administered flavonoids exhibit their pharmacological actions during inflammatory responses. Oral flavonoids could be inactivated once by a conjugation metabolism during absorption, delivered to the inflamed sites, exposed extracellularly or intracellularly to the β‐glucuronidase of resident macrophages, converted to biologically active aglycones, and modify the inflammatory response (Fig. 9). In the present study, we confirmed biological actions of flavonoid glucuronides, and that β‐glucuronidase‐possessing macrophages play a role in converting flavonoid glucuronides to active aglycones. Our study also demonstrated the actions of flavonoid glucuronides to enhance microbe phagocytosis by macrophages both in vitro and in vivo. While our study focused on adjuvant activity in innate immune cells, the inhibitory effect of flavonoids on skin inflammation may be valid and explained as well by our scenario of the deconjugation process. The present study elucidated a biologically interesting feature of glucuronides as a carrier and precursor form and would contribute to the pharmacological study of general glucuronides, not only flavonoid glucuronides. Further studies of the pharmacokinetics and metabolism of flavonoids will increase our understanding of how glucuronides exert profound effects on various tissues and diseases.

## Disclosure

Drs. Kenshi Yamasaki and Setsuya Aiba received research grant support from Tsumura & Co. Atsushi Kaneko, Takashi Matsumoto, Yosuke Matsubara, Kyoji Sekiguchi, Junichi Koseki, Ryo Yakabe, Katsuyuki Aoki, are employed by Tsumura & Co.

## Conflict of Interest

None declared.

## Supporting information

Additional supporting information may be found in the online version of this article at the publisher's web‐site.

**Figure S1**. Chemical structures of flavonoid glucuronides assayed in this study.

**Figure S2**. Representative dot‐plots of phagocytic cells in the ears treated with or without intradermal injection of FITC‐*S. aureus* particles in mice administered GEN7G or the vehicle. Particles of FITC‐conjugated and killed *S. aureus* (67 μg/10 μL/site) were injected intradermally to the ears of mice. Genistein 7‐*O*‐glucuronide (GEN7G) dissolved in saline was administrated intravenously to the mice at a dose of 1 mg/10 mL/kg immediately and 3 h post the pseudo‐infection. The ears were cut off at 6 h post the pseudo‐infection, and digested in a mixture of three kinds of enzyme (dispase I, collagenase II, and DNase I) for 2 h in 37°C, followed by preparation of single cells. Phagocytic cells were stained by anti‐mouse Ly6G (APC‐label) and anti‐mouse CD11b (PE/Cy7 label), and analyzed by flow cytometer using FACSaria II system. The cells gated in R‐1 region (CD11b^+^Ly6G^−^) and R‐2 region (CD11b^+^Ly6G^+^) of APC‐PE/Cy7 plots (A, B, and C), were designated as monocytes/macrophages and neutrophils in the present study, respectively. The intensities of FITC of the R‐1 gated‐cells (D, E, and F) and the R‐2 gated‐cells (G, H, and I) were further analyzed. The plot A, D, and G: normal mice, the plot B, E, and H: control mice treated with the pseudo‐infection and vehicle namely, the plot C, F, and I: mice treated with the pseudo‐infection and GEN7G namely. Data are indicating percent and MFI of cells in a R‐3 or R‐4 region. #*P* < 0.05 significance at Student *t*‐test.

**Figure S3**. Gene expression of estrogen receptors in RAW264.7 cells. Total RNAs were prepared from proliferating RAW264.7 cells cultured in 24‐well plates, followed by preparation of cDNAs. TaqMan gene expression assays were performed using TaqMan primers for Esr‐1 (nuclear estrogen receptor‐α), Esr‐2 (nuclear estrogen receptor‐β), and Gper1 (G protein‐coupled estrogen receptor). All data are shown as relative to a housekeeping gene, Gapdh (glyceraldehyde‐3‐phosphate dehydrogenase). The primers of these targets were purchased from ABI Biosystems (Foster City, CA). *N* = 2. ND: not detected showing 0.0001% to Gapdh.

Supporting Legends S1.

## References

[1] Surh, Y. J. 2003 Cancer chemoprevention with dietary phytochemicals. Nat. Rev. Cancer 3:768–780. 14570043 10.1038/nrc1189

[2] Grassi, D. , G. Desideri , G. Croce , S. Tiberti , A. Aggio , and C. Ferri . 2009 Flavonoids, vascular function and cardiovascular protection. Curr. Pharm. Des. 15:1072–1084. 19355949 10.2174/138161209787846982

[3] Nicolle, E. , F. Souard , P. Faure , and A. Boumendjel . 2011 Flavonoids as promising lead compounds in type 2 diabetes mellitus: molecules of interest and structure‐activity relationship. Curr. Med. Chem. 18:2661–2672. 21568900 10.2174/092986711795933777

[4] Pandey, K. B. , and S. I. Rizvi . 2009 Plant polyphenols as dietary antioxidants in human health and disease. Oxid. Med. Cell Longev. 2:270–278. 20716914 10.4161/oxim.2.5.9498 PMC2835915

[5] Beekmann, K. , L. Actis‐Goretta , P. J. van Bladeren , F. Dionisi , F. Destaillats , and I. M. Rietjens . 2012 A state‐of‐the‐art overview of the effect of metabolic conjugation on the biological activity of flavonoids. Food Funct. 3:1008–1018. 22751838 10.1039/c2fo30065f

[6] Galindo, P. , I. Rodriguez‐Gomez , S. Gonzalez‐Manzano , M. Duenas , R. Jimenez , C. Menendez , F. Vargas , J. Tamargo , C. Santos‐Buelga , F. Pérez‐Vizcaíno , et al. 2012 Glucuronidated quercetin lowers blood pressure in spontaneously hypertensive rats via deconjugation. PLoS ONE 7:e32673. 22427863 10.1371/journal.pone.0032673 PMC3299686

[7] Terao, J. , K. Murota , and Y. Kawai . 2011 Conjugated quercetin glucuronides as bioactive metabolites and precursors of aglycone in vivo. Food Funct 2:11–17. 21773581 10.1039/c0fo00106f

[8] Kroon, P. A. , M. N. Clifford , A. Crozier , A. J. Day , J. L. Donovan , C. Manach , and G. Williamson . 2004 How should we assess the effects of exposure to dietary polyphenols in vitro? Am. J. Clin. Nutr. 80:15–21. 15213022 10.1093/ajcn/80.1.15

[9] Zhang, L. , E. Angst , J. L. Park , A. Moro , D. W. Dawson , H. A. Reber , G. Eibl , O. J. Hines , V. L. Go , and Q. Y. Lu . 2010 Quercetin aglycone is bioavailable in murine pancreas and pancreatic xenografts. J. Agric. Food Chem. 58:7252–7257. 20499918 10.1021/jf101192k PMC2894579

[10] Bieger, J. , R. Cermak , R. Blank , V. C. de Boer , P. C. Hollman , J. Kamphues , and S. Wolffram . 2008 Tissue distribution of quercetin in pigs after long‐term dietary supplementation. J. Nutr. 138:1417–1420. 18641184 10.1093/jn/138.8.1417

[11] Holder, C. L. , M. I. Churchwell , and D. R. Doerge . 1999 Quantification of soy isoflavones, genistein and daidzein, and conjugates in rat blood using LC/ES‐MS. J. Agric. Food Chem. 47:3764–3770. 10552719 10.1021/jf9902651

[12] Asl, M. N. , and H. Hosseinzadeh . 2008 Review of pharmacological effects of *Glycyrrhiza* sp. and its bioactive compounds. Phytother. Res. 22:709–724. 18446848 10.1002/ptr.2362 PMC7167813

[13] Matsumoto, T. , Y. Matsubara , Y. Mizuhara , K. Sekiguchi , J. Koseki , K. Tsuchiya , H. Nishimura , J. Watanabe , A. Kaneko , K. Maemura , et al. 2015 Plasma pharmacokinetics of polyphenols in a traditional Japanese medicine, Jumihaidokuto, which suppresses *Propionibacterium acnes*‐induced dermatitis in rats. Molecules 20:18031–18046. 26437394 10.3390/molecules201018031 PMC6332076

[14] Sekiguchi, K. , J. Koseki , K. Tsuchiya , Y. Matsubara , S. Iizuka , S. Imamura , T. Matsumoto , J. Watanabe , A. Kaneko , S. Aiba , et al. 2015 Suppression of *Propionibacterium acnes*‐induced dermatitis by a traditional Japanese medicine, Jumihaidokuto, modifying macrophage functions. Evid. Based Complement Alternat. Med. 2015:439258. 26495013 10.1155/2015/439258 PMC4606168

[15] Feuerstein, R. , M. Seidl , M. Prinz , and P. Henneke . 2015 MyD88 in macrophages is critical for abscess resolution in staphylococcal skin infection. J. Immunol. 194:2735–2745. 25681348 10.4049/jimmunol.1402566

[16] Soucy, N. V. , H. D. Parkinson , M. A. Sochaski , and S. J. Borghoff . 2006 Kinetics of genistein and its conjugated metabolites in pregnant Sprague–Dawley rats following single and repeated genistein administration. Toxicol. Sci. 90:230–240. 16352619 10.1093/toxsci/kfj077

[17] Branham, W. S. , S. L. Dial , C. L. Moland , B. S. Hass , R. M. Blair , H. Fang , L. Shi , W. Tong , R. G. Perkins , and D. M. Sheehan . 2002 Phytoestrogens and mycoestrogens bind to the rat uterine estrogen receptor. J. Nutr. 132:658–664. 11925457 10.1093/jn/132.4.658

[18] Hajirahimkhan, A. , C. Simmler , Y. Yuan , J. R. Anderson , S. N. Chen , D. Nikolic , B. M. Dietz , G. F. Pauli , R. B. van Breemen , and J. L. Bolton . 2013 Evaluation of estrogenic activity of licorice species in comparison with hops used in botanicals for menopausal symptoms. PLoS ONE 8:e67947. 23874474 10.1371/journal.pone.0067947 PMC3709979

[19] Prossnitz, E. R. , and M. Barton . 2011 The G‐protein‐coupled estrogen receptor GPER in health and disease. Nat. Rev. Endocrinol. 7:715–726. 21844907 10.1038/nrendo.2011.122 PMC3474542

[20] Vivacqua, A. , D. Bonofiglio , L. Albanito , A. Madeo , V. Rago , A. Carpino , A. M. Musti , D. Picard , S. Andò , and M. Maggiolini . 2006 17Beta‐estradiol, genistein, and 4‐hydroxytamoxifen induce the proliferation of thyroid cancer cells through the g protein‐coupled receptor GPR30. Mol. Pharmacol. 70:1414–1423. 16835357 10.1124/mol.106.026344

[21] Lucki, N. C. , and M. B. Sewer . 2011 Genistein stimulates MCF‐7 breast cancer cell growth by inducing acid ceramidase (ASAH1) gene expression. J. Biol. Chem. 286:19399–19409. 21493710 10.1074/jbc.M110.195826 PMC3103318

[22] Yamazaki, S. , T. Morita , H. Endo , T. Hamamoto , M. Baba , Y. Joichi , S. Kaneko , Y. Okada , T. Okuyama , H. Nishino , et al. 2002 Isoliquiritigenin suppresses pulmonary metastasis of mouse renal cell carcinoma. Cancer Lett. 183:23–30. 12049811 10.1016/s0304-3835(02)00113-1

[23] Lim, E. K. , P. J. Mitchell , N. Brown , R. A. Drummond , G. D. Brown , P. M. Kaye , and D. J. Bowles . 2013 Regiospecific methylation of a dietary flavonoid scaffold selectively enhances IL‐1beta production following Toll‐like receptor 2 stimulation in THP‐1 monocytes. J. Biol. Chem. 288:21126–21135. 23760261 10.1074/jbc.M113.453514 PMC3774379

[24] Lee, J. , S. L. Kim , S. Lee , M. J. Chung , and Y. I. Park . 2014 Immunostimulating activity of maysin isolated from corn silk in murine RAW 264.7 macrophages. BMB Rep 47:382–387. 24286330 10.5483/BMBRep.2014.47.7.191 PMC4163854

[25] Fung, M. C. , Y. Y. Szeto , K. N. Leung , Y. L. Wong‐Leung , and N. K. Mak . 1997 Effects of biochanin A on the growth and differentiation of myeloid leukemia WEHI‐3B (JCS) cells. Life Sci. 61:105–115. 9217269 10.1016/s0024-3205(97)00365-2

[26] Takahashi, T. , M. Kobori , H. Shinmoto , and T. Tsushida . 1998 Structure‐activity relationships of flavonoids and the induction of granulocytic‐ or monocytic‐differentiation in HL60 human myeloid leukemia cells. Biosci. Biotechnol. Biochem. 62:2199–2204. 9972240 10.1271/bbb.62.2199

[27] Kawaii, S. , Y. Tomono , E. Katase , K. Ogawa , and M. Yano . 1999 Effect of citrus flavonoids on HL‐60 cell differentiation. Anticancer Res. 19:1261–1269. 10368686

[28] Campbell, L. , E. Emmerson , H. Williams , C. R. Saville , A. Krust , P. Chambon , K. A. Mace , and M. J. Hardman . 2014 Estrogen receptor‐alpha promotes alternative macrophage activation during cutaneous repair. J. Invest. Dermatol. 134:2447–2457. 24769859 10.1038/jid.2014.175

[29] Kovats, S. 2015 Estrogen receptors regulate innate immune cells and signaling pathways. Cell. Immunol. 294:63–69. 25682174 10.1016/j.cellimm.2015.01.018 PMC4380804

[30] Li, R. , Y. Shen , L. B. Yang , L. F. Lue , C. Finch , and J. Rogers . 2000 Estrogen enhances uptake of amyloid beta‐protein by microglia derived from the human cortex. J. Neurochem. 75:1447–1454. 10987824 10.1046/j.1471-4159.2000.0751447.x

[31] Kim, Y. W. , R. J. Zhao , S. J. Park , J. R. Lee , I. J. Cho , C. H. Yang , S. G. Kim , and S. C. Kim . 2008 Anti‐inflammatory effects of liquiritigenin as a consequence of the inhibition of NF‐kappaB‐dependent iNOS and proinflammatory cytokines production. Br. J. Pharmacol. 154:165–173. 18332856 10.1038/bjp.2008.79 PMC2438972

[32] Ishisaka, A. , K. Kawabata , S. Miki , Y. Shiba , S. Minekawa , T. Nishikawa , R. Mukai , J. Terao , and Y. Kawai . 2013 Mitochondrial dysfunction leads to deconjugation of quercetin glucuronides in inflammatory macrophages. PLoS ONE 8:e80843. 24260490 10.1371/journal.pone.0080843 PMC3834324

[33] Rathee, P. , H. Chaudhary , S. Rathee , D. Rathee , V. Kumar , and K. Kohli . 2009 Mechanism of action of flavonoids as anti‐inflammatory agents: a review. Inflamm. Allergy Drug Targets 8:229–235. 19601883 10.2174/187152809788681029

[34] Kim, H. P. , K. H. Son , H. W. Chang , and S. S. Kang . 2004 Anti‐inflammatory plant flavonoids and cellular action mechanisms. J. Pharmacol. Sci. 96:229–245. 15539763 10.1254/jphs.crj04003x

[35] Ganai, A. A. , A. A. Khan , Z. A. Malik , and H. Farooqi . 2015 Genistein modulates the expression of NF‐kappaB and MAPK (p‐38 and ERK1/2), thereby attenuating d‐galactosamine induced fulminant hepatic failure in Wistar rats. Toxicol. Appl. Pharmacol. 283:139–146. 25620059 10.1016/j.taap.2015.01.012

[36] Song, M. , X. Tian , M. Lu , X. Zhang , K. Ma , Z. Lv , Z. Wang , Y. Hu , C. Xun , Z. Zhang , et al. 2015 Genistein exerts growth inhibition on human osteosarcoma MG‐63 cells via PPARgamma pathway. Int. J. Oncol. 46:1131–1140. 25586304 10.3892/ijo.2015.2829

[37] Norrby, M. , A. Madej , E. Ekstedt , and L. Holm . 2013 Effects of genistein on oestrogen and progesterone receptor, proliferative marker Ki‐67 and carbonic anhydrase localisation in the uterus and cervix of gilts after insemination. Anim. Reprod. Sci. 138:90–101. 23452834 10.1016/j.anireprosci.2013.01.011

[38] LaRock, C. N. , and B. T. Cookson . 2013 Burning down the house: cellular actions during pyroptosis. PLoS Pathog. 9:e1003793. 24367258 10.1371/journal.ppat.1003793 PMC3868505

[39] Accarias, S. , G. Lugo‐Villarino , G. Foucras , O. Neyrolles , S. Boullier , and G. Tabouret . 2015 Pyroptosis of resident macrophages differentially orchestrates inflammatory responses to *Staphylococcus aureus* in resistant and susceptible mice. Eur. J. Immunol. 45:794–806. 25472006 10.1002/eji.201445098

