# Supplementary figures and images for: Glucuronides of phytoestrogen flavonoid enhance macrophage function via conversion to aglycones by β‐glucuronidase in macrophages
Source: Immun Inflamm Dis. 2017 May 8;5(3):265–79. doi: 10.1002/iid3.163 (PMC5569364; doi:10.1002/iid3.163)

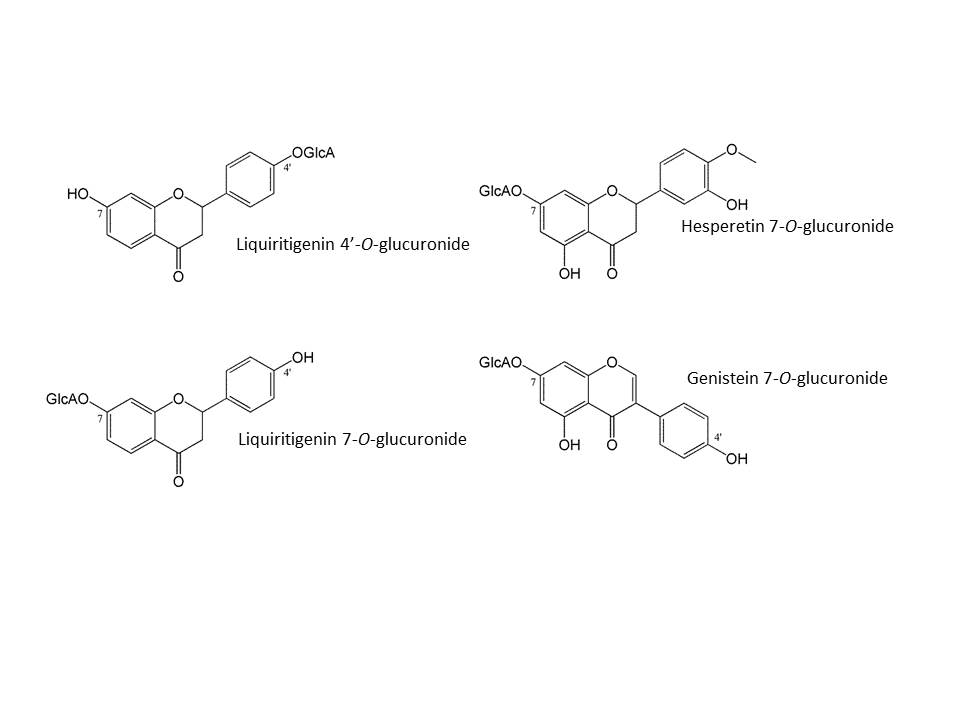

Supplement: Supplementary file 1 — Figure S1. Chemical structures of flavonoid glucuronides assayed in this study. [file IID3-5-265-s001.JPG]

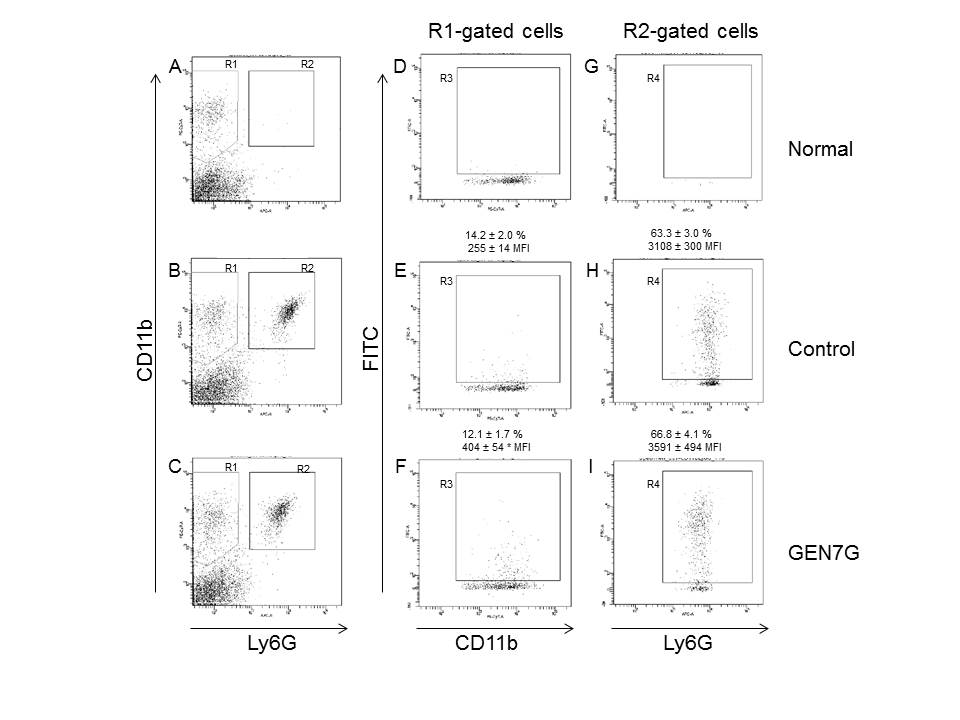

Supplement: Supplementary file 2 — Figure S2. Representative dot‐plots of phagocytic cells in the ears treated with or without intradermal injection of FITC‐S. aureus particles in mice administered GEN7G or the vehicle. Particles of FITC‐conjugated and killed S. aureus (67 μg/10 μL/site) were injected intradermally to the ears of mice. Genistein 7‐O‐glucuronide (GEN7G) dissolved in saline was administrated intravenously to the mice at a dose of 1 mg/10 mL/kg immediately and 3 h post the pseudo‐infection. The ears were cut off at 6 h post the pseudo‐infection, and digested in a mixture of three kinds of enzyme (dispase I, collagenase II, and DNase I) for 2 h in 37°C, followed by preparation of single cells. Phagocytic cells were stained by anti‐mouse Ly6G (APC‐label) and anti‐mouse CD11b (PE/Cy7 label), and analyzed by flow cytometer using FACSaria II system. The cells gated in R‐1 region (CD11b+Ly6G−) and R‐2 region (CD11b+Ly6G+) of APC‐PE/Cy7 plots (A, B, and C), were designated as monocytes/macrophages and neutrophils in the present study, respectively. The intensities of FITC of the R‐1 gated‐cells (D, E, and F) and the R‐2 gated‐cells (G, H, and I) were further analyzed. The plot A, D, and G: normal mice, the plot B, E, and H: control mice treated with the pseudo‐infection and vehicle namely, the plot C, F, and I: mice treated with the pseudo‐infection and GEN7G namely. Data are indicating percent and MFI of cells in a R‐3 or R‐4 region. #P < 0.05 significance at Student t‐test. [file IID3-5-265-s002.JPG]

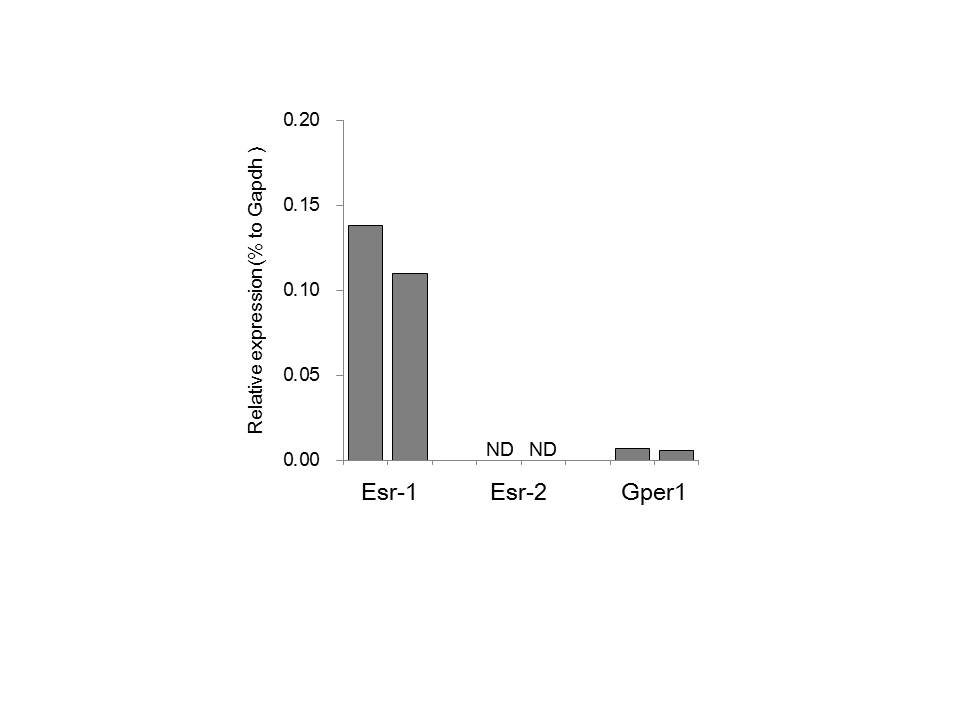

Supplement: Supplementary file 3 — Figure S3. Gene expression of estrogen receptors in RAW264.7 cells. Total RNAs were prepared from proliferating RAW264.7 cells cultured in 24‐well plates, followed by preparation of cDNAs. TaqMan gene expression assays were performed using TaqMan primers for Esr‐1 (nuclear estrogen receptor‐α), Esr‐2 (nuclear estrogen receptor‐β), and Gper1 (G protein‐coupled estrogen receptor). All data are shown as relative to a housekeeping gene, Gapdh (glyceraldehyde‐3‐phosphate dehydrogenase). The primers of these targets were purchased from ABI Biosystems (Foster City, CA). N = 2. ND: not detected showing 0.0001% to Gapdh. [file IID3-5-265-s003.JPG]
