# Supplementary material for: Glucuronides of phytoestrogen flavonoid enhance macrophage function via conversion to aglycones by β‐glucuronidase in macrophages
Source: Immun Inflamm Dis. 2017 May 8;5(3):265–79. doi: 10.1002/iid3.163 (PMC5569364; doi:10.1002/iid3.163)
Supplement: Supplementary file 4 — Supporting Legends S1. [file IID3-5-265-s004.docx]

**Supplementary Data**

**Supplementary Fig. S1. Chemical structures of flavonoid glucuronides assayed in this study.**

**Supplementary Fig. S2. Representative dot-plots of phagocytic cells in the ears treated with or without intradermal injection of FITC-*S. aureus* particles in mice administered GEN7G or the vehicle.**

Particles of FITC-conjugated and killed *S. aureus* (67 μg/10μL/site) were injected intradermally to the ears of mice. Genistein 7-*O*-glucuronide (GEN7G) dissolved in saline was administrated intravenously to the mice at a dose of 1 mg/10mL/kg immediately and 3 h post the pseudo-infection. The ears were cut off at 6 h post the pseudo-infection, and digested in a mixture of three kinds of enzyme (dispase I, collagenase II, and DNase I) for 2 h in 37°C, followed by preparation of single cells. Phagocytic cells were stained by anti-mouse Ly6G (APC-label) and anti-mouse CD11b (PE/Cy7 label), and analyzed by flow cytometer using FACSaria II system. The cells gated in R-1 region (CD11b^+^Ly6G^–^) and R-2 region (CD11b^+^Ly6G^+^) of APC-PE/Cy7 plots (A, B, C), were designated as monocytes/macrophages and neutrophils in the present study, respectively. The intensities of FITC of the R-1 gated-cells (D, E, F) and the R-2 gated-cells (G, H, I) were further analyzed. The plot A, D, G: normal mice, the plot B, E, H: control mice treated with the pseudo-infection and vehicle i.v., the plot C, F, I: mice treated with the pseudo-infection and GEN7G i.v. Data are indicating percent and MFI of cells in a R-3 or R-4 region. #: P < 0.05 significance at Student *t*-test.

**Supplementary Fig. S3. Gene expression of estrogen receptors in RAW264.7 cells.**

Total RNAs were prepared from proliferating RAW264.7 cells cultured in 24-well plates, followed by preparation of cDNAs. TaqMan gene expression assays were performed using TaqMan primers for Esr-1 (nuclear estrogen receptor-α), Esr-2 (nuclear estrogen receptor-β), and Gper1 (G protein-coupled estrogen receptor). All data are shown as relative to a housekeeping gene, Gapdh (glyceraldehyde-3-phosphate dehydrogenase). The primers of these targets were purchased from ABI Biosystems (Foster City, CA.). N = 2. ND: not detected showing 0.0001% to Gapdh.
